# The complete mitochondrial genome of Asian palm civet (*Paradoxurus hermaphroditus*) with phylogenetic consideration

**DOI:** 10.1080/23802359.2018.1443853

**Published:** 2018-02-26

**Authors:** Vaishnavi Kunteepuram, Ara Sreenivas, Wajeeda Tabasum, Anil K. Challagandla, Ajay Gaur

**Affiliations:** Laboratory for the Conservation of Endangered Species, CSIR – Centre for Cellular and Molecular Biology (CCMB), Hyderabad, India

**Keywords:** *Paradoxurus hermaphroditus*, Asian palm civet, mitochondrial genome, phylogeny, conservation

## Abstract

Asian palm civet (*Paradoxurus hermaphroditus*) is one of the smallest palm civet which is least studied. Here, we report the first complete mitochondrial (mt) genome of Asian palm civet (*P. hermaphroditus*). The circular mt genome with a length of 16,706 bp contained 1 control region, 2 rRNAs, 13 protein-coding genes, and 22 tRNAs. Overall base composition of the complete mt DNA was 33.7% A, 30.5% T, 22.9% C, and 12.9% G. All the genes in mt genome of Asian palm civet (*P. hermaphroditus*) were distributed on the H-strand, except *ND6* and eight tRNA genes encoded on the L-strand. Maximum likelihood (ML) and Bayesian inference (BI) methods were used to infer the phylogenetic relationship of *P. hermaphroditus*. The phylogenetic analysis shows that all species from the family Viverridae cluster together, in which *P. hermaphroditus* exhibits the closest relationship with *P. larvata*.

The subfamily Viverridae comprises of five genera: *Paradoxurus*, *Paguma*, *Arcticis, Arctogalidia*, and *Macrogalidia* that are found in South and Southeast Asia (Wozencraft 2005). Till date, only five complete mitochondrial genomes of viverrids have been reported (Zhang et al. 2015; Hassanin and Veron 2016; Hassanin 2016; Weng et al. 2016; Salleh et al. 2017). Here, we report the first complete mitochondrial (mt) genome sequence of Asian palm civet (*Paradoxurus hermaphroditus*, GenBank Accession No. MG200264).

Primary fibroblasts culture established as described by Yelisetti et al. (2016), from post-mortem tissue of a male adult Asian palm civet from Nehru Zoological Park, Hyderabad, India (N: 17°21′04″E: 78°26′59″) and maintained at LaCONES Genome Bank (LGB-PC-001), was used for DNA extraction by standard phenol-chloroform isoamyl alcohol method (Sambrook et al. 1989). PCR amplification and sequencing of the entire mt genome was done using three pairs of primers with an average amplicon size of 5–6 kb each.

The total length of the *P. hermaphroditus* mt DNA was 16,706 bp. It contained 1 control region (D-loop), 2 rRNA genes, 13 protein-coding genes, and 22 tRNA genes. Origin of reading frame of all protein-coding genes and gene orders were identical to other members of Viverridae. The overall base composition of A, T, G, and C was 33.7%, 30.5%, 12.9%, and 22.9%, respectively and the GC content of the mt genome was 35.8%. Most of the *P. hermaphroditus* genes were encoded on the H-strand, except for *ND6* gene and eight tRNA genes (*tRNA^Gln^*, *tRNA^Ala^*, *tRNA^Asn^*, *tRNA^Cys^*, *tRNA^Tyr^*, *tRNA^Ser^*, *tRNA^Glu^*, *tRNA^Pro^*). The control region was located between *tRNA^Pro^* and *tRNA^Phe^*. The length of D-loop was 1321 bp ranging from 15,386–16,706 bp. Most of the protein-coding genes had ATG (Met) as start codon except *ND2*, *ND3*, and *ND5* (ATT). Further, *ND1*, *ATP8*, *ND4*, *Cyt b* ended with TAG; *COX1*, *COX2*, *ATP6*, *ND5*, and *ND6* ended with TGA, *ND2*, *ND3*, *ND4l*, and *COX3* ended with TAA as stop codons.

For sequence comparison, mt genomes of five species of viverrids were obtained from NCBI GenBank. The mt genome of Asiatic lion (*Panthera leo persica*, Felidae, Tabasum et al. 2016) was used as an outgroup. The phylogenetic relationships were constructed among all viverrids based on 13 protein-coding genes. The best model of sequence evolution for each gene in both the analysis was obtained by jModeltest 2.1.5 (Darriba et al. 2012). Bayesian analysis (BI) was performed using Mr Bayes v 3.2 (Ronquist et al. 2012) with four chains of 1.1 × 10^5^ generations and sampling the trees every 100 generations. Maximum-likelihood (ML) analysis was performed using MEGA 7 (Kumar et al. 2016).

All species of Viverridae clustered together, and *P. hermaphroditus* exhibited the closest relationship with *P. larvata* (Figure 1) in consistence with previous reports (Patou et al. 2010, Veron et al. 2015, 2017). Since, there is a limited genetic data available on Asian palm civet (*P. hermaphrodites)* from Indian sub-continent, the present study will contribute significantly to in-depth phylogenetic studies and proper assessment of conservation status of this species.

**Figure 1.**
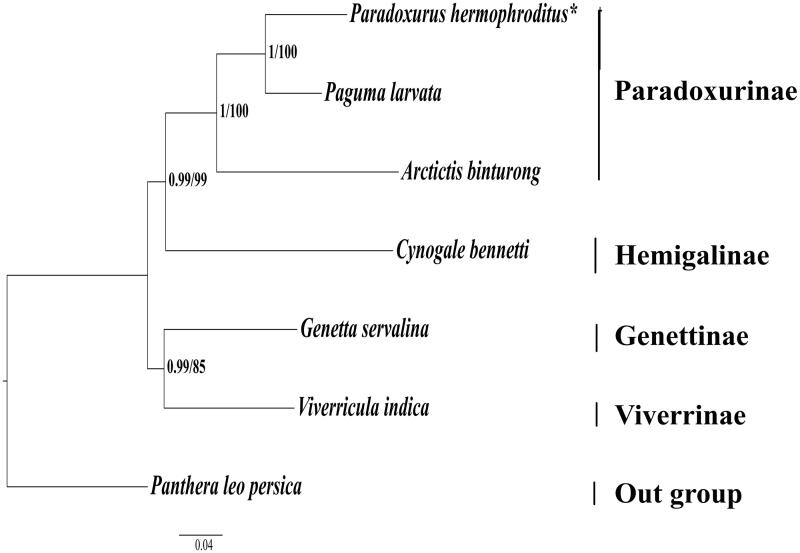
Phylogenetic relationships among the Viverrids. Clade support values are given by Bayesian posterior probabilities/ML bootstrap percentages. The phylogenetic tree was rooted using *P. leo persica* (KU234271). The analyzed species and their respective NCBI accession number are as follows: *Paguma larvata* (NC_029403), *Arctictis binturong* (KY117560), *Cynogale bennetii* (KY117544), *Genetta servalina* (NC_024568), and *Viverricula indica* (NC_025296). *P. hermaphroditus (MG200264 – this study).

## Nucleotide sequence accession number

The complete mitogenome sequence of Asian palm civet (*Paradoxurus hermaphroditus*) has been assigned Genbank accession number MG200264.
